# Dual Blockade of PI3K and EGFR Pathways by Flavonoids from *Idesia polycarpa* Maxim Cake Meal: Valorization of Agro-Industrial Waste for NSCLC Therapy

**DOI:** 10.3390/foods14183278

**Published:** 2025-09-22

**Authors:** Zhenyu Yang, Kai Luo, Dan Chen, Lei Dou, Xiufang Huang, Jianquan Kan

**Affiliations:** 1 College of Biological and Food Engineering, Hubei Minzu University, Enshi 445000, China; zhenyuyang9291@163.com (Z.Y.); luokai_79@163.com (K.L.); doulei0119@163.com (L.D.); 2 Hubei Provincial Key Laboratory of Occurrence and Intervention of Rheumatic Diseases, Hubei Minzu University, Enshi 445000, China; rc520627@163.com; 3 Hubei Provincial Clinical Medical Research Center for Nephropathy, Enshi 445000, China; 4 College of Food Science, Southwest University, Chongqing 400715, China

**Keywords:** agro-food by-products, *Idesia polycarpa* Maxim, flavonoids, A549 cells, anti-NSCLC activity, PI3K/AKT pathways, EGFR-MAPK pathways

## Abstract

Efficient utilization of food industry waste supports sustainable development. *Idesia polycarpa* Maxim cake meal (an oil-processing by-product) is rich in bioactive flavonoids, but the refined purification, anti-non-small cell lung cancer (NSCLC) activity, and mechanism of its total flavonoids (IPTF) remain unclear—restricting high-value use. This study optimized IPTF purification via polyamide resin gradient elution and characterized its chemical composition by HPLC/LC-MS. In vitro assays assessed IPTF’s effects on A549 cell proliferation, migration, invasion, colony formation, and apoptosis; network pharmacology and molecular docking predicted mechanisms, validated via Western blotting for key signaling pathways. Results showed IPTF purity was significantly improved after purification; HPLC/LC-MS identified rutin, quercetin, and six minor components as key constituents. IPTF inhibited A549 proliferation, and network pharmacology indicated it synergistically targets the PI3K/AKT and EGFR-MAPK pathways—validated by reduced phosphorylation of p-AKT, p-EGFR, and p-ERK. This work offers a novel strategy for *I. polycarpa* cake meal valorization and highlights IPTF’s potential as a multi-target natural candidate for NSCLC therapy.

## 1. Introduction

Driven by globalization and population growth, the food industry generates substantial food waste, which raises socio-ethical, environmental, and economic concerns [1]. Within the grains and oils sector, oil crop processing yields considerable by-products such as cake meals. Despite their nutritional value, these materials remain underutilized industrially [2]. Consequently, the food industry is actively pursuing high-value-added products from waste streams to facilitate circular economy models [3].

*Idesia polycarpa* Maxim. (commonly known as oil grape), a deciduous tree of the Flacourtiaceae family, represents a significant woody oil crop in China [4]. Its fruit contains up to 43.6% oil, rich in unsaturated fatty acids [5,6], its cake meal—the primary processing residue following oil extraction via pressing—constitutes over 60% of the fruit’s dry mass. Currently primarily repurposed as animal feed or compost, this utilization strategy is neither sustainable nor environmentally sound [7]. Consequently, valorizing *Idesia polycarpa* cake meal to enhance its comprehensive utilization value is critical for advancing high-value transformation of agricultural and food industry waste. Recent studies have identified functional components in the cake meal, including antioxidant peptides [8] and polysaccharides [9]. Flavonoids attract considerable research interest due to their broad biological activities (For instance, epicatechin influences skeletal muscle function in aged mice [10], quercetin inhibits proliferation in various cancer cells [11], and rutin can alleviate oxidative stress [12]). Notably, despite detected bioactive flavonoids in the fruits [13], research on flavonoid constituents in the cake meal remains scarce. Significant knowledge gaps persist regarding systematic characterization of flavonoid composition, content quantification, purification process optimization, and particularly systematic evaluation of bioactivities (e.g., anti-NSCLC effects) and underlying mechanisms. These limitations substantially impede comprehensive exploitation and value-added utilization of this abundant resource.

Lung cancer is the top cause of global cancer deaths [14]. Non-small cell lung cancer (NSCLC) represents the predominant subtype, with only approximately 15% of patients diagnosed at early stages, while the majority (84%) present with advanced disease upon initial diagnosis [15]. Recent advances have led to novel therapies like immunotherapy and targeted therapy (widely used in oncology) [16], and identifying actionable targets (e.g., EGFR, PI3K/AKT/mTOR, RAS-MAPK) has revolutionized advanced NSCLC treatment [17]. However, long-term use of targeted agents causes toxicities (e.g., hepatotoxicity, nephrotoxicity) that require dose reduction or discontinuation, impairing efficacy [18]. Given this context, natural products have emerged as pivotal resources for anticancer drug discovery due to their multi-target mechanisms and favorable safety profiles [19,20]. Phytochemicals may overcome current therapeutic limitations by synergistically modulating multiple pathways, potentiating efficacy while mitigating dose-dependent toxicity.

Network pharmacology accelerates drug discovery by constructing biomolecular interaction networks to clarify disease-compound-target relationships and systematically predict multi-target drug interactions [21], relying on databases with extensive experimental data and algorithm-predicted chemical components/targets [22]. Although traditional methods for studying herbal medicine efficacy—via systematic workflows (extraction–isolation–purification–structural elucidation–experimental validation)—yield reliable conclusions, their time-consuming and inefficient nature necessitates methodological innovation [23]. In contrast, network pharmacology enhances efficiency by overcoming these limitations through multi-target prediction and biological network integration—thus, this study uses a combined strategy of network pharmacology prediction and experimental validation for cross-verification.

In preliminary work, we optimized an environmentally friendly ultrasound-assisted extraction (UAE) protocol using single-factor experiments combined with response surface methodology (RSM), successfully recovering IPTF from cake meal. Subsequent purification via macroporous resin achieved initial enrichment, while LC-MS/MS characterized the principal flavonoid constituents [24]. Building on this, we used polyamide resin gradient elution to refine IPTF, with HPLC and LC-MS enabling precise quantification of bioactive compounds to establish a standardized high-value utilization process. To further explore IPTF’s pharmaceutical potential, this study integrated network pharmacology and molecular docking to systematically predict its anti-NSCLC targets and pathways. Functional validation was performed in A549 cells via proliferation (CCK-8, colony formation), migration, and invasion assays, supplemented by flow cytometry for cell cycle and apoptosis analysis; mechanistic insights were clarified via Western blotting (WB). This comprehensive strategy—covering extraction, quantification, bioactivity, and mechanism research—provides robust support for using IPTF from oil-processing waste as an anti-NSCLC agent.

## 2. Materials and Methods

### 2.1. Materials

IPTF was extracted from Idesia polycarpa defatted meal. The *Idesia polycarpa* fruits were sourced from Lichuan City, Hubei Province, China, with the cake meal obtained as a by-product during laboratory-scale oil extraction. A549 cells were provided by Hubei Provincial Key Laboratory of Occurrence and Intervention of Rheumatic Diseases, Hubei Minzu University (Hubei, China), and A549 cells (CL-0016) were kindly provided by Wuhan Pricella Biotechnology Co., Ltd. The fully automated flow cytometer (SH880S) was purchased from Sony (Tokyo, Japan). A multimode microplate reader was acquired from Thermo Fisher Scientific (Shanghai, China). An inverted fluorescence microscope was obtained from Olympus (Tokyo, Japan). Dimethyl sulfoxide (DMSO), CCK-8 assay kits, cisplatin, and Matrigel matrix were procured from Beyotime Biotechnology (Beijing, China). PBS and DMEM medium were sourced from Bkmamlab (Changde, China). Fetal bovine serum (FBS) was supplied by Zhejiang Tianhang Biotechnology Co., Ltd. (Hangzhou, China). Penicillin-streptomycin (P/S) solution, trypsin solution, and Annexin V-FITC/PI apoptosis detection kits were purchased from Biosharp Life Sciences (Hefei, China). Rutin (≥98% purity) was acquired from Yuanye Bio-Technology (Shanghai, China).

### 2.2. Purification of IPTF

The subsequent purification procedure comprised three stages: (a) Defatting: The concentrated extract was mixed with hexane (1:1, *v*/*v*), magnetically stirred at 1500 rpm for 1 h, and allowed to stand for phase separation, followed by discarding the upper lipid phase. (b) Solvent extraction: Residual hexane was removed via rotary evaporation. The aqueous phase pH was adjusted to 3.5 using 1 M HCl, then extracted with ethyl acetate (5:1, *v*/*v*) under 1500 rpm stirring for 1 h. After phase separation, the organic layer was collected, concentrated, and stored at −80 °C. (c) Column chromatography: The ethyl acetate fraction was lyophilized for 48 h, reconstituted in 20% ethanol (2 mg/mL), and loaded onto a polyamide resin (80-mesh) column at 1.5 mL/min. Stepwise gradient elution was performed with 0%, 35%, and 55% ethanol. The 55% ethanol fraction was collected and lyophilized to yield purified IPTF.

The total flavonoid content of IPTF was quantified using the aluminum nitrate-sodium nitrite colorimetric method (NaNO_2_-Al(NO_3_)_3_-NaOH system). A standard curve was established with rutin reference standards at gradient concentrations (0–100 μg/mL). Linear regression yielded the equation: Y = 0.0019X − 0.0021 (X: rutin concentration, μg/mL; Y: absorbance), demonstrating excellent linearity (R^2^ = 0.9994). For purity determination, 0.1 g of IPTF was dissolved in 70% ethanol, volumetrically adjusted to 50 mL, filtered through a 0.22 μm membrane, and subjected to absorbance measurement via the aforementioned colorimetric method. The flavonoid content was calculated using the following formula:

(1) Purity(%)=(C0×V×F)/M0×100%

where *C*0 is the total flavonoid concentration in *Idesia polycarpa* cake meal (in mg/mL), *V* is the volume of the solvent (in mL), *F* is the dilution factor, *M*0 is the mass of the sample (in mg).

### 2.3. Quantitative Analysis Based on HPLC and LC-MS

Quantitative analysis of various flavonoid components in IPTF was performed using the external standard method. Each standard was accurately weighed, and 10 mg/mL individual standard stock solutions were prepared in methanol; subsequently, an appropriate amount was mixed to prepare a 1 μg/mL mixed standard working solution for subsequent use. Standard curves with concentration gradients of 1, 10, 50, 100, 150, 200, 300, 400, and 500 ng/mL were established for instrumental analysis. Chromatographic analysis was performed using a Thermo Vanquish ultra-high-performance liquid chromatography system equipped with a Waters HSS T3 column (50 × 2.1 mm, 1.8 μm). Mobile phase A was ultrapure water containing 0.1% formic acid, and phase B was acetonitrile solution containing 0.1% formic acid. The flow rate was 0.3 mL/min, column temperature was 40 °C, and injection volume was 2 μL. The elution gradient program was as follows: 0–2 min maintained at A:B = 90:10 (*v*/*v*); 2–6 min linearly ramped to A:B = 40:60 (*v/v*); 6–9 min maintained at 40:60 (*v*/*v*); 9.1 min reverted to 90:10 (*v/v*) and held until 12 min. Mass spectrometric detection was conducted using a Thermo Q Exactive high-resolution mass spectrometer, with an electrospray ionization (ESI) source in negative ion mode and Full scan-ddMS^2^ scanning mode. Parameters were set as follows: sheath gas flow rate 40 arb, auxiliary gas flow rate 10 arb, ion spray voltage −2800 V, ion source temperature 350 °C, capillary temperature 320 °C, and full scan range of primary mass spectrometry *m*/*z* 100–900. All experiments in this section were performed by Shanghai Yansu Technology Co., Ltd. (Shanghai, China; www.shiyanjia.com (accessed on 7 May 2025)).

### 2.4. Network Pharmacology

#### 2.4.1. Prediction of Potential Targets of IPTF and Non-Small Cell Lung Cancer

Qualitative and quantitative analysis results were integrated and imported into the TCMSP database (https://www.tcmsp-e.com/tcmsp.php (accessed on 6 May 2025)) to screen for active components of IPTF. The screening criteria were set as oral bioavailability (OB) ≥ 30% and drug-likeness (DL) ≥ 0.18, yielding the core active components: quercetin, epicatechin, and morin. Subsequently, human targets (Homo sapiens) were retrieved for these compounds using PubChem (https://pubchem.ncbi.nlm.nih.gov/; accessed 9 May 2025) and SwissTargetPrediction (http://swisstargetprediction.ch/; accessed 9 May 2025). Target names were standardized and duplicates were removed via UniProt (https://www.uniprot.org/; accessed 11 May 2025), generating compound-target interaction data. NSCLC targets were acquired from GeneCards (https://www.genecards.org/; accessed 11 May 2025) using “non small cell lung cancer” as the search query. Shared targets between IPTF and NSCLC were identified through Venn diagram analysis using Venny 2.1 (https://bioinfogp.cnb.csic.es/; accessed 11 May 2025).

#### 2.4.2. Protein–Protein Interaction (PPI) Network Construction

Common targets were imported into STRING 12.0 (https://string-db.org/; accessed 12 May 2025) with Homo sapiens specified as the organism to construct the PPI network and perform functional enrichment analysis. The interaction data (minimum required interaction score: 0.7) were exported to Cytoscape 3.9.1 for topological analysis using the CytoNCA plugin. Core targets were identified by applying the following criteria: Degree Centrality (DC), Betweenness Centrality (BC) and Closeness Centrality (CC) ≥ median. A visualized PPI network was generated based on these core targets with node size proportional to DC value and edge thickness representing interaction confidence.

#### 2.4.3. Gene Ontology (GO) and Kyoto Encyclopedia of Genes and Genomes (KEGG) Pathway Enrichment

The intersection targets were submitted to the DAVID database (https://davidbioinformatics.nih.gov/ (accessed on 13 May 2025)) for GO functional annotation and KEGG pathway enrichment analysis. A significance threshold of *p* < 0.05 was set (statistically significant). Results were visualized according to the following rules: For GO enrichment, the top 10 terms ranked by enrichment significance in biological process (BP), molecular function (MF), and cellular component (CC) were selected to generate bar charts; for KEGG enrichment, the top 20 pathways ranked by enrichment significance were chosen to construct bubble plots.

### 2.5. Molecular Docking

The top 6 core targets ranked by topological analysis in the PPI network were selected as receptors, and the core active components of IPTF were used as ligands. The 3D crystal structures of receptor proteins were retrieved from the PDB database, and ligand molecular structures were downloaded from the TCMSP database in MOL2 format. Receptors were preprocessed using AutoDockTools 1.5.7: hydrogen atoms were added, and crystallographic water molecules were removed; after preprocessing, receptor-ligand molecular docking was performed. Docking results were quantitatively evaluated using binding energy (unit: kcal/mol), where negative values indicate binding affinity. Finally, the 3D structure visualization of docking complexes was achieved using PyMOL 3.1.1 (New York, NY, USA).

### 2.6. Cellular Assays

#### 2.6.1. Cell Line Selection and Culture

The human non-small cell lung cancer cell line A549 was selected for this study. Cells were maintained in high-glucose DMEM supplemented with 10% FBS and 1% penicillin-streptomycin antibiotic solution. Cultures were incubated at 37 °C in a humidified atmosphere containing 5% CO_2_. All experiments utilized T-25 flasks seeded at 5 mL medium per flask.

#### 2.6.2. Effect of IPTF on A549 Cell Viability

Cell viability was assessed using the CCK-8 assay with modifications to Jiang et al. [25]. Briefly, exponentially growing A549 cells were counted with a hemocytometer and seeded in 96-well plates at 4 × 10^5^ cells/well. After 24 h incubation, the medium was replaced with 100 μL fresh medium containing serially diluted IPTF. Subsequently, 20 μL of CCK-8 solution (final concentration 10% *v/v*) was added to each well; after 2 h of incubation, absorbance was measured at 450 nm using a microplate reader. Cell viability was calculated using the following equation:

(2) Cell survival rate(%)=(A1−A2)/(A3−A4)×100%

where *A*1 denotes the absorbance of wells containing both cells and sample, *A*2 denotes the absorbance of wells containing sample without cells, *A*3 denotes the absorbance of wells containing cells without sample, and *A*4 denotes the absorbance of blank wells (no cells, no sample).

#### 2.6.3. Wound-Healing Assay

The procedure was adapted from that reported by Wang et al. [26] with minor modifications. A549 cells were seeded in 6-well plates at 1 × 10^5^ cells/well and incubated until 90% confluency. A sterile 200 μL pipette tip (autoclaved) was then used to introduce a single vertical scratch in the confluent monolayer of each well. After medium replacement with IPTF-containing medium, images of the scratches were captured at 0, 12, 24, 36 and 48 h using an inverted microscope. The scratch healing rate was calculated using the following equation:

(3) Scratch healing rate(%)=(1−St/S0)×100%

where *S*0 represents the wound area at 0 h and *St* the corresponding area at the indicated time points.

#### 2.6.4. Colony-Formation Assay

Exponentially growing A549 cells were seeded in 6-well plates at 2000 cells/well (2 mL medium/well) and incubated overnight. The medium was then replaced with fresh complete medium containing serial dilutions of IPTF, and cells were further incubated for 48 h. After medium removal, cells were washed twice with 2 mL PBS, fixed with 4% paraformaldehyde for 10 min at room temperature, washed again with PBS, and stained with 0.1% crystal violet solution for 10 min at room temperature. Excess stain was removed by gentle rinsing with distilled water, and plates were air-dried at room temperature. Colonies were imaged at 20× magnification using an inverted microscope (three random fields per well) and counted with ImageJ 1.8.0 software.

#### 2.6.5. Transwell Invasion Assay

The assay was adapted from the protocol described by Zhang et al. [27] with minor modifications. Frozen Matrigel was thawed overnight at 4 °C, and Transwell inserts were pre-chilled at 4 °C. On ice, Matrigel was mixed 1:8 (*v/v*) with serum-free medium; 100 μL of this mixture was then added to each Transwell upper chamber and polymerised for 4 h at 37 °C, after which excess gel was removed. Each insert was rehydrated with 100 μL serum-free medium for 30 min at 37 °C. Following 4 h serum starvation, A549 cells were harvested and resuspended at 4 × 10^5^ cells/mL in serum-free medium; 100 μL of this suspension was seeded into the upper compartment of each insert. The lower chamber received 500 μL medium supplemented with 20% FBS, while 100 μL serum-free medium containing serial dilutions of IPTF was added to the upper chamber, followed by 48 h incubation at 37 °C. After incubation, non-invading cells on the upper surface were gently removed with a sterile cotton swab, and inserts were rinsed with PBS. Cells adhering to the underside of the membrane were fixed by immersing the inserts in 700 μL of 4% paraformaldehyde for 20 min at room temperature, rinsed once with PBS. Staining was performed with 0.1% crystal violet for 20 min at room temperature, followed by gentle rinsing with distilled water and air-drying. Invaded cells were imaged at 20× magnification under an inverted microscope (three random fields per membrane) and enumerated using ImageJ 1.8.0 software. The invasion inhibition rate was calculated using the following formula:

(4) Invasion inhibition rate(%)=(1−N1/N0)×100%

where *N*0 is the number of invaded cells in the treated group and *N*1 is the number of invaded cells in the vehicle control group.

#### 2.6.6. Flow-Cytometric Analysis of Apoptosis

A549 cells were seeded in 6-well plates at 2 × 10^5^ cells/well and incubated overnight. The medium was then replaced with fresh complete medium containing serial dilutions of IPTF, followed by 48 h incubation under the same conditions. Cells were harvested with EDTA-free trypsin, pelleted by centrifugation, washed twice with cold PBS, and resuspended; the resulting suspension was passed through sterile gauze to obtain a single-cell suspension. Cells were subsequently double-stained with Annexin V-FITC and propidium iodide (PI) according to the manufacturer’s instructions and analysed by flow cytometry within 30 min of staining to quantify the apoptotic rate.

#### 2.6.7. Western Blotting Analysis

After 48 h treatment with IPTF-containing medium, the medium was aspirated and cells were washed three times with ice-cold PBS. A lysis buffer was freshly prepared by mixing RIPA reagent with 10× protease and phosphatase inhibitor cocktail at a 500:1 (*v/v*) ratio. Lysis buffer (150 μL per well) was added, and cells were lysed on ice for 5 min before being scraped into microcentrifuge tubes and incubated on ice for an additional 30 min. Samples were centrifuged at 12,000 r/min for 5 min at 4 °C, and the supernatants (total protein extracts) were collected. Protein samples were denatured at 98 °C for 10 min in a heat block, cooled to room temperature, and stored at −80 °C. Proteins were resolved by SDS-PAGE using precast gels (12%) at a constant voltage of 80 V for 1 h. Separated proteins were transferred onto PVDF membranes at 130 V for 14–16 min, blocked with 5% (*w/v*) non-fat milk in TBST for 1 h, and incubated overnight at 4 °C with primary antibodies. Membranes were subsequently incubated with appropriate HRP-conjugated secondary antibodies for 1 h at room temperature in the dark. After three 5-min washes with TBST, the membranes were processed for chemiluminescence. Protein bands were visualised using a chemiluminescence imaging system and quantified by densitometry with ImageJ software. All experiments in this section were performed by Shanghai Yansu Technology Co., Ltd. (Shanghai, China; www.shiyanjia.com (accessed on 18 July 2025)).

### 2.7. Statistical Analysis

All experiments, unless otherwise specified, were conducted in triplicate. Statistical analyses were performed using GraphPad Prism 10.1.2 and Origin 2024. All data were analyzed by two-way ANOVA, with Tukey’s test employed to identify significant differences (*p* < 0.05).

## 3. Results

### 3.1. Further Purification of IPTF

#### 3.1.1. Sequential Solvent Extraction of IPTF

Building upon the pre-optimized ultrasound-assisted extraction protocol (ultrasonic power: 400 W, duration: 70 min, liquid-to-material ratio: 50:1 mL/g), crude IPTF extract (purity: 39.06 ± 0.99%) was initially obtained through AB-8 macroporous resin purification, with LC-MS/MS identifying its principal flavonoids: quercetin, quercetin-3-O-rutinoside, morin, quercetin-3-O-glucoside, epicatechin, piceid, and paeoniflorin-3-O-β-galactoside.

**Figure 1:**
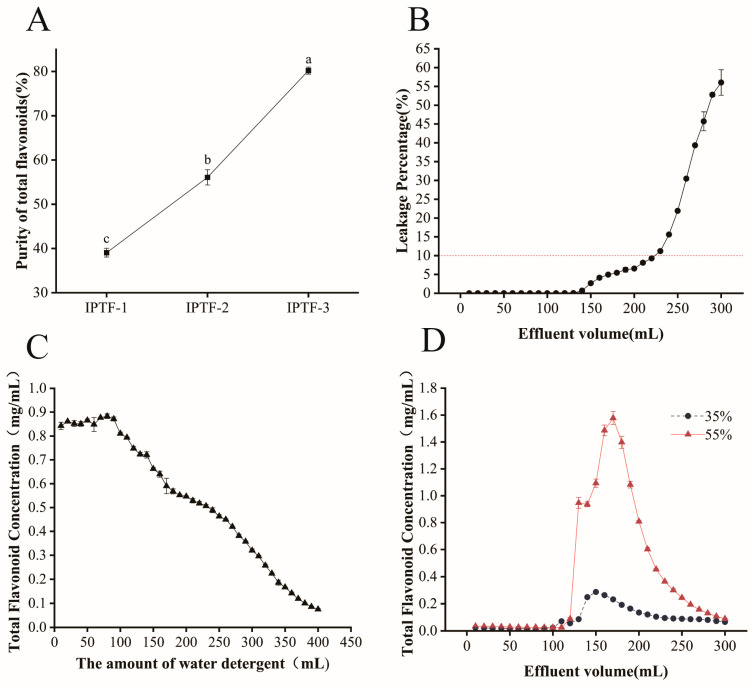
Dynamic Adsorption and Desorption of IPTF on Polyamide Resin Column. (**A**) Comparison of purity before and after purification. IPTF-1: Extract treated with AB-8 macroporous resin; IPTF-2: Extract partitioned with hexane and ethyl acetate; IPTF-3: Extract treated with polyamide resin. In the figure, different lowercase letters indicate significant differences among groups. (**B**) Leakage curves. (**C**) The amount of water detergent. (**D**) Dynamic Gradient Desorption Profile.

In our previous work, crude IPTF extracted from *Idesia polycarpa* cake meal was subjected to preliminary purification on AB-8 macroporous resin; however, this step failed to remove the bulk of the lipid fraction. Therefore, a sequential hexane–ethyl acetate extraction protocol was implemented, and the results are summarised in Figure 1A. Following sequential extraction, the total flavonoid content of IPTF rose significantly from 39.06 ± 0.99% to 56.09 ± 1.73% (*p* < 0.05). Consistent with literature reports, hexane efficiently extracts lipids [28], whereas the principal bioactive constituents of IPTF—quercetin, rutin and related flavonoid glycosides—exhibit high solubility and retained bioactivity in ethyl acetate [29]. These findings corroborate the selective roles of hexane and ethyl acetate in purifying flavonoid-rich fractions.

#### 3.1.2. Dynamic Adsorption on Polyamide Resin

Polyamide resin exhibits selective adsorption toward flavonoids and is widely employed for their separation from crude extracts [30,31]. Accordingly, hexane/ethyl acetate-pretreated IPTF was loaded onto a polyamide column for further purification under dynamic conditions. The resulting breakthrough curve reflects the resin’s adsorption capacity; the breakthrough point was defined as the effluent absorbance reaching 10% of the initial loading absorbance, indicating saturation. As shown in Figure 1B, leakage remained 0% for effluent volumes between 0 and 130 mL, demonstrating efficient adsorption of IPTF by the resin within this range. Between 130 and 230 mL, leakage gradually increased, indicating partial desorption of flavonoids. Beyond 230 mL, leakage consistently exceeded 10%, signifying complete saturation of the resin. Consequently, 230 mL was selected as the maximum loading volume for IPTF.

After breakthrough, some unbound flavonoids remained in the column; these were eluted with distilled water, simultaneously removing water-soluble impurities that might compromise IPTF purity. As depicted in Figure 1B, flavonoid concentration in the effluent decreased progressively with increasing wash volume; at 400 mL, the concentration approached 0 mg/mL, indicating near-complete removal of unbound material. Thus, 400 mL of distilled water was designated as the 0% ethanol fraction in the subsequent gradient elution.

Following elution with 400 mL of 0% (*v/v*) ethanol, stepwise desorption was performed with 35%, 55% and 75% (*v/v*) ethanol solutions. As shown in Figure 1D, the 35% ethanol fraction yielded effluent flavonoid concentrations below 0.4 mg/mL throughout; a minor fluctuation was observed between 100 and 200 mL, after which the concentration approached zero, confirming that this ethanol concentration elicits only negligible desorption of the target flavonoids. No flavonoids were detected in the subsequent 75% ethanol fraction, underscoring the selective elution of flavonoids by 55% ethanol from the polyamide resin. The freeze-dried product obtained from the 55% ethanol eluate was analysed for total flavonoid purity, with results presented in Table 1. Following polyamide column chromatography and gradient ethanol elution, the total flavonoid purity of IPTF increased significantly from 56.09 ± 1.73% to 80.16 ± 0.66% (*p* < 0.05). Among them, with cake meal as the raw material, the yield of IPTF-1 was 26.598 ± 0.058%; with IPTF-1 as the raw material, the yield of IPTF-2 was 18.097 ± 0.880%; and with IPTF-2 as the raw material, the yield of IPTF-3 was 7.867 ± 0.252%. These findings corroborate the synergistic mechanism of gradient elution: low ethanol concentrations exhibit limited desorption efficacy, whereas incremental increases in concentration markedly enhance the purity of the target compounds [32]. Consequently, elution volumes of 200 mL and 300 mL were selected for the 35% and 55% ethanol fractions, respectively.

### 3.2. Quantitative Characterisation of IPTF by HPLC and LC–MS

**Figure 2:**
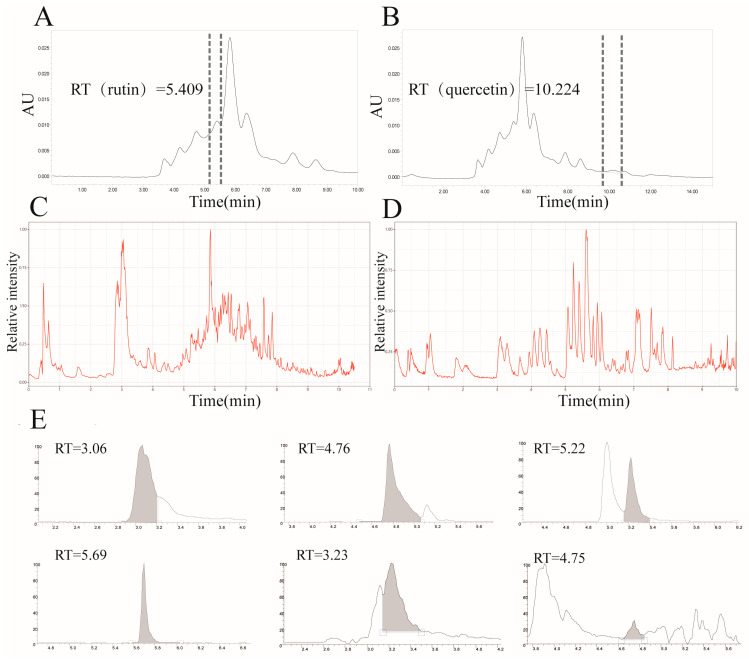
Quantitative Analysis of Flavonoid Constituents in IPTF by HPLC and LC-MS. (**A**) Content of Rutin in IPTF Analyzed by HPLC. (**B**) Content of Quercetin in IPTF Analyzed by HPLC. (**C**) Total Ion Chromatogram (TIC) of IPTF by LC-MS. (**D**) Total Ion Chromatogram (TIC) of Reference Standards by LC-MS. (**E**) Chromatogram of Target Constituents in IPTF by LC-MS: Protocatechualdehyde, Dihydromyricetin, p-Hydroxycinnamic Acid, Quercetin 3-β-D-glucoside, Phthalic Acid, and Epicatechin. Among them, the shaded area indicates the target constituents.

Based on previous evidence that quercetin and rutin are the principal flavonoids in IPTF, we quantified these compounds by external-standard HPLC (Figure 2B,C). Rutin and quercetin contents were 17.578 ± 0.213 mg/g and 2.078 ± 0.096 mg/g (DW), respectively. Notably, several unidentified peaks in the HPLC chromatograms suggested additional minor constituents, but their identity and abundance could not be ascertained owing to insufficient sensitivity. To comprehensively profile the chemical composition of IPTF, we employed ultra-high-performance liquid chromatography coupled to a Q-Exactive high-resolution mass spectrometer (UPLC-Q-Exactive/MS). Dual-criteria confirmation—retention-time deviation < 0.2 min and mass accuracy < 10 ppm—enabled absolute quantification of trace constituents. Database matching identified six minor compounds (Figure 2C–E): protocatechualdehyde, dihydromyricetin, p-hydroxycinnamic acid, quercetin-3-β-D-glucoside, phthalic acid and epicatechin (Table 1). This integrated approach markedly enhanced both the accuracy and comprehensiveness of trace-level analysis in a complex matrix, providing a robust chemical foundation for subsequent bioactivity studies.

### 3.3. Network Pharmacology Analysis

#### 3.3.1. Identification of Core Targets

**Figure 3:**
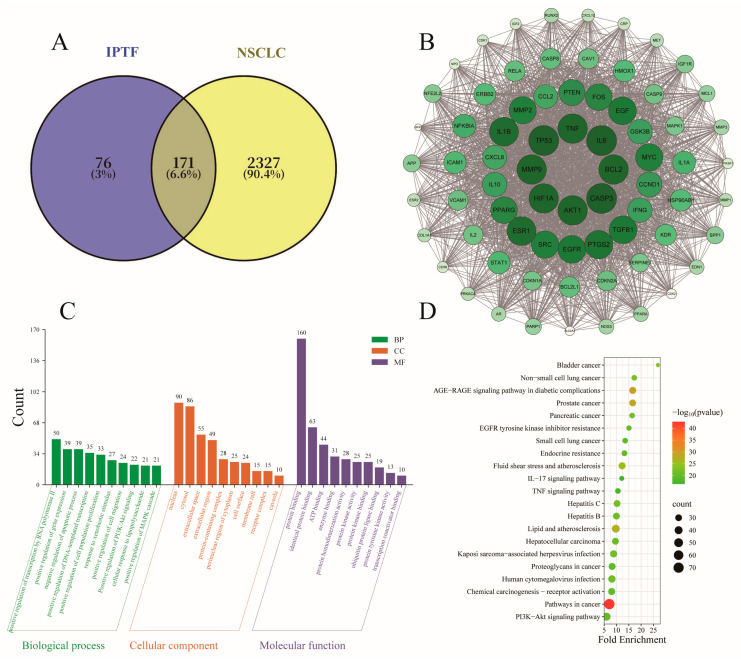
(**A**) Venn diagram of compound targets in IPTF and anti-NSCLC targets. (**B**) PPI network of 72 intersection anti-NSCLC targets. (**C**) the bar graph of the top 10 GO terms, including biological process (BP), cellular compound (CC), and molecular function (MF). (**D**) the top 20 significantly enriched pathways by KEGG enrichment analysis.

Network pharmacology is a systems-biology-based approach that integrates network analysis to dissect drug–target interactions and predict underlying mechanisms of action [33]. Using the SwissTargetPrediction and TCMSP databases, 247 unique targets associated with the active constituents of IPTF were identified after merging and deduplication, while 2498 NSCLC-related targets were retrieved from SwissTargetPrediction. Intersection analysis via Venny 2.1 yielded 171 shared targets between disease and active ingredients (Figure 3A).

To elucidate interactions among potential targets, the 171 common targets were submitted to STRING 12.0 for PPI network analysis. The resulting PPI network comprised 171 nodes and 4290 edges. This topological complexity implies that IPTF’s bioactive constituents may exert anti-NSCLC effects through multi-target synergistic mechanisms across diverse pathways. Topological analysis was subsequently performed with the CytoNCA plug-in, yielding median Degree Centrality (DC), Betweenness Centrality (BC) and Closeness Centrality (CC) values of 42, 45.081 and 0.567, respectively. After screening, a total of 72 nodes met the criteria of DC, BC, and CC ≥ median values; therefore, these 72 targets were regarded as key targets (Figure 1B), and the key proteins were imported into Cytoscape 3.9.1 software for visualization. Proteins with higher Degree Centrality values are more centrally located in the network with larger nodes, indicating that the target is more important in the interaction network. Therefore, the 72 key targets were ranked according to their Degree Centrality values, and the top 6 targets were selected as core targets, namely: Cellular tumor antigen p53 (TP53), AKT serine/threonine kinase 1 (AKT1), Tumor necrosis factor (TNF), Interleukin-6 (IL6), Caspase-3 (CASP3), and Epidermal growth factor receptor (EGFR). Among them, AKT1 serves as a pivotal downstream effector of the PI3K pathway, orchestrating apoptosis inhibition, proliferative signalling and metabolic regulation [34]. EGFR, a receptor tyrosine kinase of the ErbB family, is a well-established therapeutic target in NSCLC [35]. Ligand binding induces homo- or hetero-dimerisation, activating the kinase domain; autophosphorylation then propagates downstream cascades that govern cell survival, proliferation and differentiation [36]. The co-selection of AKT1 and EGFR as core targets suggests that IPTF may contribute to suppressing NSCLC progression by synergistically regulating the PI3K/AKT and EGFR-MAPK signaling ways.

#### 3.3.2. GO and KEGG Enrichment Analysis of Key Targets

GO and KEGG enrichment analyses were performed for IPTF and 171 shared targets. A total of 867 biological process, 92 cellular component, and 223 molecular function terms were obtained from the GO analysis. The terms were ranked by *p*-value, and the top 10 terms in each category that were significantly associated with NSCLC were visualized (Figure 3C). In the BP category, the most significant term was “positive regulation of transcription mediated by RNA polymerase II” (50 genes), suggesting that IPTF may extensively inhibit the transcriptional activation of oncogenes. Additionally, the enrichment of “positive regulation of the PI3K/AKT signaling pathway” (22 genes), “positive regulation of the MAPK signaling pathway” (21 genes), and “negative regulation of apoptosis” (39 genes) indicates that IPTF may induces cancer cell death by synergistically regulating pro-survival signals and relieving apoptosis inhibition. The CC analysis showed that the target proteins are mainly localized in the nucleus (90 genes) and cytoplasm (86 genes). Enrichment in the nucleus implies interference with transcriptional complexes, while the association with lipid rafts (15 genes) and caveolae (10 genes) suggests that it may affect the spatial organization of membrane receptor signal transduction. At the MF level, the target genes are highly enriched in protein binding (160 genes) and kinase-related functions. The significance of protein kinase activity (25 genes) and kinase binding (31 genes) provides a structural basis for IPTF to inhibit the phosphorylation of kinases such as EGFR and AKT.

In the KEGG analysis, visualization of the top 20 pathways ranked by *p*-value (Figure 3D) showed that the PI3K-AKT signaling pathway (*p* = 1.96 × 10^−20^) and non-small cell lung cancer pathway (*p* = 1.26 × 10^−22^) were significantly enriched. Together with the GO analysis, this confirms that the inhibition of the PI3K/AKT-MAPK dual pathways is the core mechanism of action of IPTF.

### 3.4. Molecular Docking of Key Anti-NSCLC Compounds with Core Targets

Molecular docking is a structure-based computational method that generates the binding pose and affinity between ligands and targets [37]. To investigate the mechanism of action of key anti- NSCLC compounds, AutoDockTools 1.5.7 was used to calculate the binding energy between core compounds and core targets, with docking parameters set to 10 independent runs to obtain the optimal binding conformation. According to the literature criteria [38], a binding energy of ≤−5 kcal/mol indicates good ligand-receptor binding. The results (Table 2) showed that the three core anti-NSCLC compounds had good affinity with all six key targets (binding energy < −5 kcal/mol); among them, epicatechin exhibited strong binding activity with TP53 and EGFR (binding energy < −7 kcal/mol). Notably, studies by Priyanka Maiti et al. have confirmed that epicatechin derived from Vernonia cinerea can effectively inhibit EGFR-mutant lung cancer [39], which is consistent with the results of this study, suggesting that epicatechin as a potential key compound in IPTF that may taeget EGFR.

**Figure 4:**
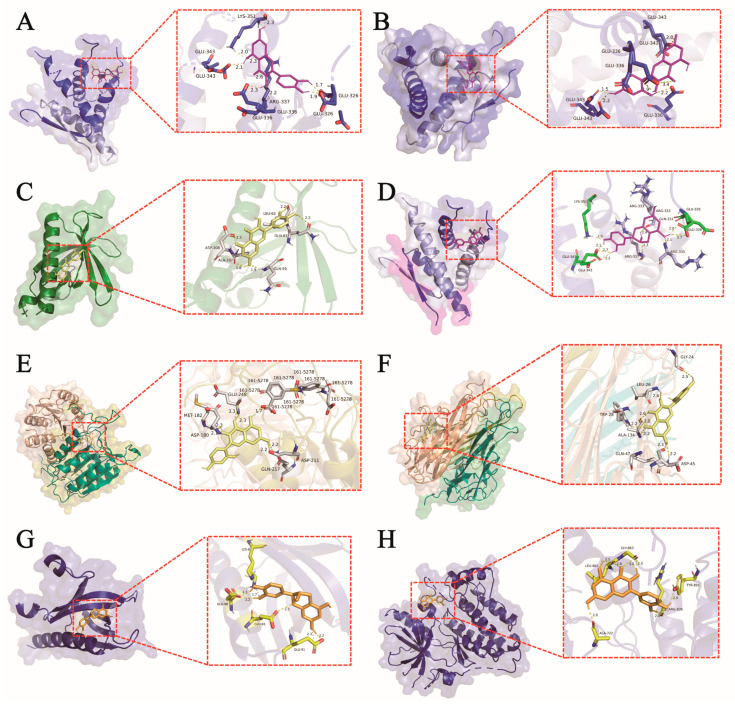
The molecular docking results of core target with active compounds of IPTF. Molecular Docking between (**A**) Quercetin and TP53. (**B**) Morin and TP53. (**C**) Quercetin and AKT1. (**D**) Epicatechin and TP53. (**E**) Morin and CASP3. (**F**) Morin and TNF. (**G**) Epicatechin and AKT1. (**H**) Quercetin and EGFR. Active compounds are represented by ball-and-stick model, the secondary structure of the protein was represented by ribbon. The yellow dotted liners represent hydrogen bond.

For each anti-NSCLC compound, the top three complexes with the optimal binding energy to key targets were selected, and the 3D structures of the docked complexes were visualized using PyMOL 3.1.1. The results indicated that the three types of anti-NSCLC compounds primarily bind to target proteins through hydrogen bond interactions, among which the amino acid residues Glu343, Glu336, Glu326, Lys351, and Arg333 exhibit the highest binding frequency. Furthermore, Quercetin, Morin, and Epicatechin can bind at the same target sites (e.g., Glu343) (Figure 4A,B,D). Notably, Hydrogen bonds play a crucial role in drug design, and the number of hydrogen bonds is closely related to the binding affinity [40]. The binding sites of quercetin with AKT1 involve Leu62, Gln59, and Ala107, with hydrogen bond distances of 2.2 Å, 1.9 Å, and 1.8 Å, respectively (Figure 4C), indicating significant conformational stability [41]. Relevant studies have shown that AKT1, as a core target for NSCLC, forms a stable binding interaction with quercetin, and induces apoptotic effects by downregulating AKT1 [42]. This further suggests that IPTF may inhibits NSCLC progression, potentially via regulation of the PI3K/AKT signaling pathway through quercetin, which is consistent with the results predicted in Section 3.1.1 and Section 3.1.2. Combining the results of this study, it can be concluded that the regulation of the PI3K/AKT and EGFR-MAPK signaling pathways by the three anti-NSCLC compounds in IPTF is one of the mechanisms underlying the anti-NSCLC activity of IPTF.

### 3.5. Effects of IPTF on the Viability and Migratory Capacities of A549 Cells

**Figure 5:**
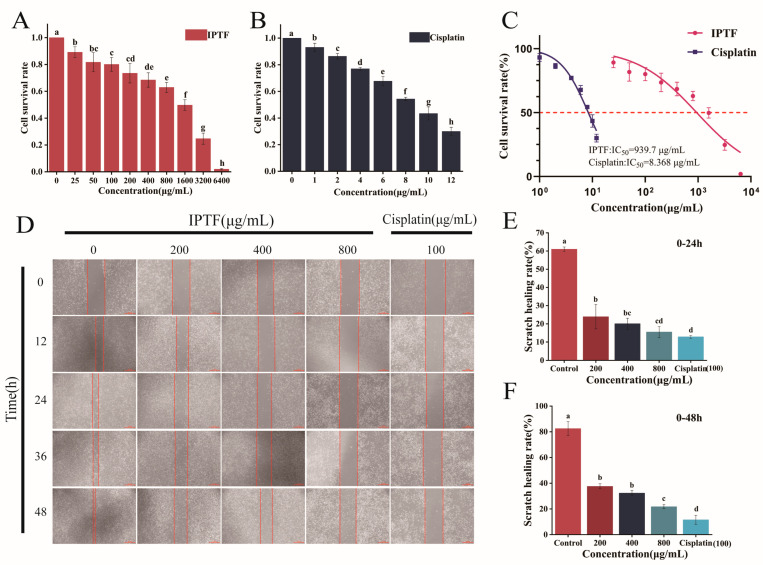
Effects of IPTF on cell viability, IC_50_ values, and wound healing in A549 cells. (**A**) Cell viability after IPTF treatment. (**B**) Cell viability after cisplatin treatment. (**C**) Presents the IC_50_ values of IPTF and cisplatin. (**D**) Results of the wound-healing assay of A549 cells treated with IPTF. (**E**) Effect of IPTF on wound-healing rate of A549 cells within 0–24 h. (**F**) Effect of IPTF on wound-healing rate of A549 cells within 0–48 h. In the figure, different lowercase letters indicate significant differences among groups.

Cisplatin is a primary drug for the treatment of NSCLC, and cisplatin-based therapeutic regimens can achieve partial or complete tumor regression in NSCLC patients [43]. To quantitatively evaluate the effect of IPTF on A549 cell proliferation, cisplatin was used as a positive control, and the CCK-8 assay was employed to assess the impact of different concentrations of IPTF on A549 cell proliferation. The results, as shown in Figure 5A, demonstrated that in the low concentration range (25–100 μg/mL), cell viability remained above 85% with weak inhibitory activity. When the concentration increased to the medium-high range (200–1600 μg/mL), cell viability decreased in a gradient manner (approximately 70% at 400 μg/mL and 50% at 1600 μg/mL). In the high concentration range (3200–6400 μg/mL), the inhibitory effect increased sharply (cell viability decreased to 30% at 6400 μg/mL). This result is consistent with the inhibitory trend of dihydromyricetin on A549 cell proliferation reported by Cheng et al. [44]. Among these, calculated by the fitting equation, the IC_50_ values of IPTF and cisplatin were 939.7 μg/mL and 8.368 μg/mL, respectively, within the 0–48 h period (Figure 5C). The above results fully demonstrate that the inhibitory effect of IPTF on A549 cell proliferation is confirmed. In subsequent experiments, 200 μg/mL, 800 μg/mL, and 1400 μg/mL were selected as the experimental concentrations of IPTF.

To avoid interference of the inhibitory effect of IPTF on cell proliferation with cell migration ability, 200 μg/mL, 400 μg/mL, and 800 μg/mL were selected as the concentrations for this experimental group. The cell migration assay showed that after 24 h of IPTF treatment, the migration ability of A549 cells was significantly inhibited (*p* < 0.05) (Figure 5D). When the IPTF concentration increased to 400 μg/mL, the migration inhibition rate only decreased by 8.7 ± 1.2% (*p* > 0.05), indicating that the inhibitory effect of the medium concentration on migration was limited. However, the inhibitory effect significantly increased again at higher concentrations (*p* < 0.05). After 48 h of treatment, the inhibitory effect of all concentration groups weakened, confirming that IPTF exhibits better inhibitory activity within 24 h. The above results verify that IPTF impairs the lateral movement ability of A549 cells in a time-concentration-dependent manner.

### 3.6. Effects of IPTF on the Invasive and Clonogenic Capacities of A549 Cells

**Figure 6:**
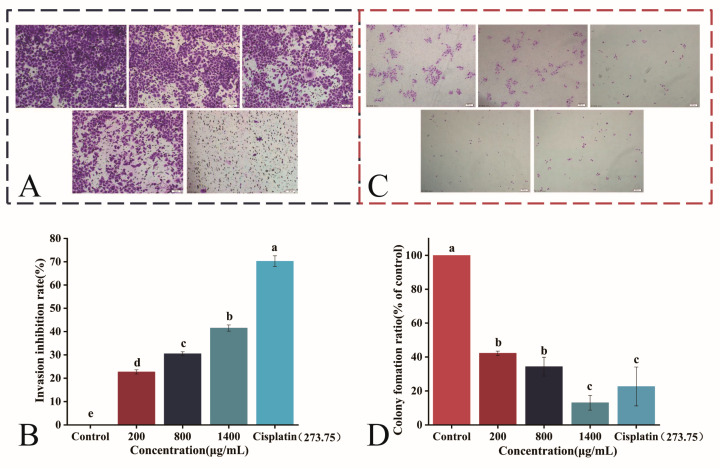
Crystal violet staining results. (**A**) The transwell invasion assay was used to detect the effect of IPTF. (**B**) Results of the invasion inhibition rate of A549 cells treated with IPTF. (**C**) Shows the results of A549 cells, respectively, representing the microscopic images of IPTF-treated A549 cells. (**D**) Results of the colony formation rate of A549 cells. In the figure, different lowercase letters indicate significant differences among groups.

The Transwell invasion assay uses Matrigel to simulate the extracellular matrix environment. Cells subjected to serum-free starvation treatment need to penetrate the Matrigel-coated polycarbonate membrane into the lower chamber, thereby simulating the invasion process of A549 cells. The cell invasion assay was performed to further investigate the effect of IPTF on the invasive ability of A549 cells. The results, as shown in Figure 6A, indicated that in the blank control group, a large number of cells successfully invaded into the lower chamber, confirming good cell viability. However, after treatment of cells with 200 μg/mL IPTF, the number of invading cells in the lower chamber significantly decreased (*p* < 0.05) (Figure 6B), with obvious blank areas observed in the microscopic field of view. Furthermore, with increasing IPTF concentration, the invasive ability of A549 cells significantly decreased, showing a good dose-dependent manner. This trend of change is consistent with the results reported by Shi et al. [45], thereby confirming that IPTF has a significant inhibitory effect on the invasion of A549 cells.

The colony formation of A549 cells was detected to further evaluate the effect of IPTF on the clonogenic ability of A549 cells. The results, as shown in Figure 6C, demonstrated that in the blank control group, large colonies (≥100 cells/colony) were formed within 48 h, with the number reaching 160 ± 21, indicating vigorous cell proliferation. In contrast, the number of colonies in the 200 μg/mL IPTF treatment group significantly decreased to 68 ± 2 (*p* < 0.05), with reduced colony size (≤70 cells/colony). With increasing concentration, the number and size of colonies further decreased, and only 21 ± 7 colonies remained when the IPTF concentration reached 1400 μg/mL. Notably, there was no statistical difference in the inhibitory effect between IPTF at this concentration and cisplatin (273.75 μg /mL) (*p* > 0.05) (Figure 6D), suggesting that high-concentration IPTF can produce a clonogenic inhibitory effect comparable to that of the positive control drug. In summary, the inhibitory effect of IPTF on colony formation of A549 cells is confirmed, and this result is mutually corroborated with the cell viability assay results in Section 3.5, collectively confirming the proliferation-inhibiting activity of IPTF.

### 3.7. Analysis of the Effect of IPTF on Apoptosis of A549 Cells Based on Flow Cytometry

**Figure 7:**
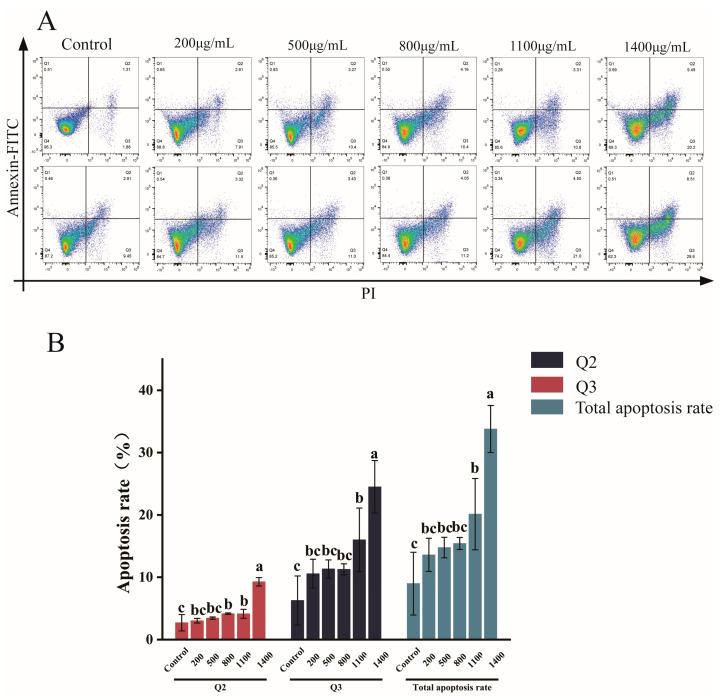
(**A**) Representative dot plot of flow cytometry analysis of cells treated with different concentrations of IPTF (cells were double-stained with Annexin V-FITC/ PI after 48 h treatment). Q1 represents the percentage of necrotic cells, Q2 represents the percentage of late-apoptotic cells, Q3 represents the percentage of early-apoptotic cells, Q4 represents the percentage of live cells. (**B**) Apoptosis rate under different concentrations of IPTF treatment, in which total apoptosis rate = Q2 + Q3. In the figure, different lowercase letters indicate significant differences among groups.

Apoptosis is one of the mechanisms that inhibit cell growth. To verify whether IPTF can induce apoptosis in A549 cells, flow cytometry was used in this study to detect the effect of IPTF on cell apoptosis, and the results are shown in Figure 7A. The results showed that compared with the control group, the apoptosis rate of A549 cells gradually increased after treatment with gradient concentrations of IPTF for 48 h. Among them, under the induction of high-concentration IPTF, the apoptosis rate significantly increased, showing a good dose-dependent increase (*p* < 0.05). This result is consistent with the trend of apoptosis in B16F0 cells induced by potato protein (PPI) reported by Li et al. [46], fully supporting the potential apoptotic-inducing effect of IPTF on A549 cells.

### 3.8. Western Blot Validation of the Regulatory Effect of IPTF on the PI3K/AKT and EGFR-MAPK Signaling Pathways

**Figure 8:**
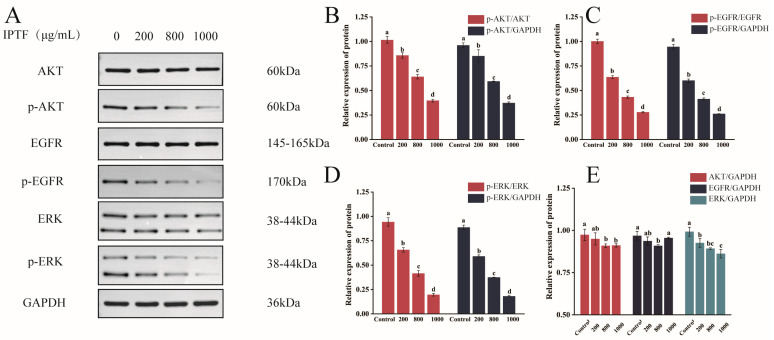
IPTF decreased phosphorylation and activation of PI3K/AKT and EGFR-MAPK signaling pathways. (**A**) Effects of IPTF on expression of proteins from PI3K/AKT and EGFR-MAPK signaling pathway in A549 cells. (**B**) Quantitative analysis of p-AKT/AKT ratio after IPTF treatment. (**C**) Quantitative analysis of p-EGFR/EGFR ratio after IPTF treatment. (**D**) Quantitative analysis of p-ERK/ERK ratio after IPTF treatment. (**E**) Expression levels of AKT/GAPDH, EGFR/GAPDH, and ERK/GAPDH after IPTF treatment. In the figure, different lowercase letters indicate significant differences among groups.

Based on the prediction from KEGG enrichment analysis in Section 3.3.2, which suggested that IPTF may inhibit non-small cell lung cancer progression by regulating the PI3K/AKT and EGFR-MAPK signaling pathways, this study used GAPDH as an internal reference and validated this mechanistic hypothesis via Western blotting, with the results shown in Figure 8. The results showed that with increasing concentrations of IPTF treatment, the phosphorylation levels of key signaling molecules in A549 cells exhibited significant dose-dependent inhibition, and the ratios of p-AKT/AKT (Figure 8B), p-EGFR/EGFR (Figure 8C), and p-ERK/ERK (Figure 8D) all significantly decreased (*p* < 0.05), indicating that IPTF can effectively block the phosphorylation activation of AKT (at Ser473) and EGFR/ERK (at Tyr1068/Tyr204). Notably, under the same treatment conditions, the expression levels of total proteins AKT, EGFR, and ERK relative to the internal reference GAPDH did not show a significant dose-dependent decrease (Figure 8E), indicating that the regulatory effect of IPTF on pathway proteins specifically occurs at the post-translational modification level rather than through reducing protein synthesis. This is consistent with the research results reported by Sun et al. [47], confirming the molecular mechanism by which IPTF may synergistically represses the malignant proliferation phenotype of A549 cells by simultaneously regulating the PI3K/AKT pathway (regulating cell survival) and the EGFR-MAPK cascade pathway (regulating proliferation signal transduction).

## 4. Discussion

The rapid development of the food industry is accompanied by the generation of large quantities of by-products. Their improper disposal not only causes resource waste but also poses severe environmental challenges. As the main waste from woody oil processing, *Idesia polycarpa* cake meal has a dry matter content accounting for more than 60% of the total fruit mass. Traditionally, it is only used as feed or compost raw material, resulting in low resource utilization efficiency and limited economic added value.

Macroporous resin adsorption is a purification method using adsorbents. It utilizes the differences between adsorbent resins and substances; the properties of functional groups on the surface of adsorbent particles, such as polarity, acidity-basicity, and hydrogen bonding capacity, affect the adsorption selectivity and behavior [48]. However, substances obtained by preliminary purification using macroporous resins contain a large number of impurities (e.g., lipids), which hinders the study of their physiological activities. Therefore, further separation and purification are required to remove these impurities and improve the purity of the target compounds. In the present work, sequential extraction with hexane-ethyl acetate effectively removed fat-soluble impurities, increasing the flavonoid purity from 39.06 ± 0.99% to 56.09 ± 1.73%, which demonstrates the advantage of solvent polarity differences in the selective extraction of flavonoids. Polyamide resin achieves further efficient separation through specific hydrogen bonding between its amide groups and the phenolic hydroxyl groups of flavonoids. Dynamic adsorption and desorption experiments showed that the 55% ethanol elution fraction had the optimal desorption efficiency for target flavonoids, which is consistent with the characteristic of flavonoid glycosides that hydrogen bonding is weakened and solubility is increased at this ethanol concentration. In summary, by optimizing the sequential solvent extraction (hexane/ethyl acetate) combined with polyamide resin gradient elution process, the purity of IPTF was increased from 39.06 ± 0.99% to 80.16 ± 0.66%, and its purification efficiency exceeded that of the traditional macroporous resin method. This strategy significantly improves the resource conversion efficiency of agricultural waste, not only reducing environmental pollution pressure but also conforming to the concept of circular economy development.

Lung cancer is a major malignant tumor threatening human health and a leading cause of cancer-related deaths. Although chemotherapy, as a standard treatment, has improved the prognosis of NSCLC patients, tumor drug resistance remains one of the major obstacles in lung cancer treatment. This study focuses on the IPTF extracted and purified from *Idesia polycarpa* cake meal waste, aiming to evaluate its anti-NSCLC potential. Using HPLC combined with LC-MS, we accurately quantified the main flavonoid components in IPTF, such as rutin (17.578 ± 0.213 mg/g) and quercetin (2.078 ± 0.096 mg/g), and successfully identified 6 trace active substances, including protocatechuic aldehyde, dihydromyricetin, and epicatechin. This provides a solid basis for elucidating its chemical basis and subsequent mechanistic studies. Notably, studies have confirmed that these identified compound monomers or their structural analogs exhibit favorable anti-NSCLC activity in both in vitro and in vivo models [49,50]. Based on this chemical basis and literature-supported activity expectations, this study systematically evaluated the inhibitory effect of IPTF on the human non-small cell lung cancer A549 cell line. The key cell proliferation inhibitory effects were demonstrated by the CCK-8 assay, colony formation assay, migration and invasion ability determination, and apoptosis rate measurement via flow cytometry. These results collectively suggest that IPTF may effectively block the proliferation process of A549 cells, potentially through mechanisms that may primarily involve the induction of apoptosis and suppression of metastatic behaviors. However, the experimental results in this section raise a critical concern: the effective concentration of IPTF for inhibiting A549 cell proliferation is relatively high (IC_50_ = 939.7 μg/mL). While this observation is consistent with a common limitation of in vitro studies on complex natural extracts, there remains a pivotal question regarding whether such a concentration can be achieved in vivo, particularly at tumor sites. In clinical practice, cisplatin is administered intravenously, and its pharmacokinetic properties support its therapeutic utility: it rapidly reaches a peak plasma concentration (Cmax) of approximately 2.6 μg/mL within minutes of administration (time to maximum concentration, Tmax ≈ 1 min) [51]. Even though its concentration declines rapidly thereafter, this rapid and significant increase in concentration is crucial for its anti-tumor efficacy. In stark contrast, the key flavonoid components of IPTF (e.g., quercetin) typically exhibit oral bioavailability of <10% [52]. Given that oral administration is the most plausible route for natural product-based interventions, this characteristic poses a critical constraint. Clinical pharmacokinetic data further underscore this challenge: even after high-dose oral administration of quercetin, the reported peak plasma concentration of free (bioactive) quercetin in humans is only approximately 369.20 ng/mL. More notably, quercetin absorption is not only slow (Tmax ≈ 4 h) but also incomplete [53], which further reduces the likelihood of maintaining sufficient concentrations at tumor sites to exert anti-tumor effects.

Network pharmacology, as a powerful research method, integrates the essence of systems biology, bioinformatics, and computational network science. It can analyze the complex molecular interaction network between drugs and the human body at the system level, providing insights into comprehensive pharmacological mechanisms. To systematically explore the potential molecular mechanism by which IPTF inhibits NSCLC, this study integrated network pharmacology approaches. KEGG pathway enrichment analysis showed that the targets of the core active components of IPTF were highly significantly enriched in key signaling pathways such as the PI3K-AKT signaling pathway (*p* = 1.96 × 10^−20^) and the non-small cell lung cancer pathway (*p* = 1.26 × 10^−22^). Existing studies have fully confirmed that signaling pathways such as PI3K-AKT play a core regulatory role in tumorigenesis and development [54]. These pathways do not operate independently but synergize with each other through complex crosstalk, forming a dynamic regulatory network that collectively influences disease progression. Based on PPI network analysis, we identified that AKT1 and EGFR are key core targets of IPTF for anti-NSCLC activity. AKT1 is a crucial downstream effector kinase in the PI3K/AKT/mTOR signaling pathway. This pathway is one of the classical pathways that regulate cell proliferation, survival, apoptosis, and metabolism [55]. When PI3K is stimulated and activated, it translocates to the cell membrane and phosphorylates phosphatidylinositol 3,4-bisphosphate (PIP2) to phosphatidylinositol 3,4,5-trisphosphate (PIP3) [56]. PIP3 then acts as a second messenger to bind to AKT, leading to the phosphorylation of the Thr308 site and its activation. Activated AKT then phosphorylates numerous downstream targets (e.g., mTOR, BAD, etc.). Among them, the activated mTOR signaling pathway regulates protein synthesis and cell growth [57], and the regulation of protein production and cell growth further modulates cell proliferation, migration, invasion, and apoptosis. Meanwhile, epidermal growth factor receptor (EGFR, also known as HER1 and ErbB1) is one of the earliest receptor tyrosine kinases (RTKs) recognized as anti-cancer targets. Notably, EGFR is not only a key switch that drives its own downstream EGFR-MAPK pathway (i.e., the RAS-RAF-MEK-ERK cascade) but also one of the most important common upstream activators of the PI3K/AKT pathway. When EGFR binds to its ligand, it undergoes dimerization and autophosphorylation of tyrosine residues (e.g., key sites such as Tyr1068) [58]. Its phosphorylated tyrosine residues can serve as docking sites to recruit adapter proteins containing SH2 domains. Among them, the recruitment of the Grb2-SOS complex leads to the activation of the small G protein RAS, which in turn initiates the RAF-MEK-ERK (MAPK) cascade [59,60], regulating cell proliferation and differentiation. Meanwhile, phosphorylated EGFR can also directly or indirectly (e.g., through adapter proteins such as Gab1) recruit the p85 regulatory subunit of PI3K, thereby activating PI3K and its downstream AKT signaling pathway [61]. This structure enables EGFR to act as a hub that connects and synergistically activates the two core signaling pathways: PI3K/AKT and MAPK. On this basis, we validated the molecular mechanism of IPTF against NSCLC through molecular docking experiments and Western blot assays. Further Western blot results showed that IPTF treatment could effectively inhibit the phosphorylation levels of key phosphorylation sites in A549 cells. Therefore, it can be preliminarily confirmed that IPTF synergistically represses the malignant proliferation phenotype of A549 cells by simultaneously inhibiting the PI3K/AKT pathway (regulating cell survival) and the EGFR-MAPK cascade pathway (regulating proliferation signal transduction).

This study successfully optimized the purification process of IPTF, significantly improving the purity and yield of the target product. Future studies can further focus on in-depth analysis of the complex component system of IPTF, isolate and identify more monomeric components, and systematically evaluate their respective structure–activity relationships and synergistic effects to more comprehensively clarify its pharmacodynamic material basis. Mechanistic studies have shown that IPTF effectively represses the malignant proliferation phenotype of non-small cell lung cancer A549 cells by synergistically inhibiting two key signaling pathways: PI3K/AKT and EGFR-MAPK. However, this profound disparity between IPTF’s in vitro effective concentration and the achievable in vivo concentrations of its key components highlights a significant translational barrier for its potential clinical application. To address this limitation, future work must adopt a dual focus: first, confirming IPTF’s in vivo anti-NSCLC efficacy using animal models to validate whether pharmacologically relevant doses can inhibit tumor progression; second, strategically overcoming its pharmacokinetic limitations. Prospective strategies include developing advanced drug delivery systems (e.g., nanoparticle carriers, phospholipid complexes) to enhance the solubility, stability, and targeted delivery of IPTF’s active constituents—thereby improving their bioavailability and tumor accumulation. Concurrently, rigorous evaluations of systemic toxicity (e.g., hepatotoxicity, nephrotoxicity) at achievable in vivo doses are essential to ensure safety.

## 5. Conclusions

This study successfully established an efficient purification process for IPTF. Through optimization of polyamide resin chromatography, a high-purity extract (80.16 ± 0.66%) was obtained. Based on the combined use of HPLC and LC-MS, the core components rutin (17.578 ± 0.213 mg/g) and quercetin (2.078 ± 0.096 mg/g) were accurately quantified, and 6 trace active components such as protocatechuic aldehyde were identified, comprehensively elucidating its chemical basis. In the non-small cell lung cancer A549 cell model, IPTF exhibited significant dose-dependent anti-tumor activity, specifically manifested as inhibiting cell proliferation (IC_50_ = 939.7 μg/mL), impairing migration and invasion abilities, and inducing cell apoptosis. By integrating network pharmacology prediction and molecular docking technology, this study is the first to reveal the core mechanism by which IPTF exerts anti-cancer effects through the synergistic regulation of the PI3K/AKT and EGFR-MAPK dual signaling pathways. This prediction was rigorously verified by Western blot experiments: IPTF can specifically inhibit the activation of key phosphorylation sites of AKT (Ser473), EGFR (Tyr1068), and ERK (Tyr204), thereby synergistically blocking the malignant proliferation process of tumor cells. This study not only provides an innovative path for the resource utilization of agricultural waste but also reveals the development potential of *Idesia polycarpa*-derived flavonoids as multi-target anti-NSCLC candidate drugs.

## Figures and Tables

**Table 1:** Quantitative Chromatogram of Target Constituents in IPTF by LC-MS.

NO.	Compounds	Content (ng/mg)	Retention Time	Expected Mass (*m*/*z*)	Detected Mass
1	Protocatechualdehyde	246.465	3.0595973	137.02442	137.0243225
2	Dihydromyricetin	174.259	4.7581958	319.04594	319.0458984
3	p-Hydroxycinnamic Acid	172.885	5.2209085	163.04007	163.0399323
4	Quercetin 3-β-D-glucoside	89.687	5.6933109	463.0882	463.0877991
5	Phthalic acid	55.817	3.2304703	165.01933	165.0191345
6	Protocatechualdehyde	246.465	4.7581958	137.02442	137.0243225

**Table 2:** Binding energy between core targets and key anti-NSCLC compounds.

NO.	Key Proteins	PBD ID	Compounds		
			Quercetin	Morin	Epicatechin
			Binding Energy (kcal/mol)		
1	TP53	8UQR	−6.85	−6.25	−7.30
2	CASP3	1NMS	−5.49	−5.96	−5.89
3	IL6	1ALU	−5.66	−5.85	−6.26
4	TNF	1A8M	−5.39	−6.37	−6.75
5	AKT1	1UNQ	−6.06	−5.56	−6.80
6	EGFR	2RGP	−6.24	−5.45	−7.05

## Data Availability

The data presented in this study are available on request from the corresponding authors due to privacy restrictions.

## Abbreviations

The following abbreviations are used in this manuscript:

IPTF	Total flavonoids from *Idesia polycarpa* Maxim cake meal
HPLC	High-performance liquid chromatography
LC-MS	Liquid chromatography-mass spectrometry
LC-MS/MS	Liquid chromatography-tandem mass spectrometry
NSCLC	Non-small cell lung cancer
GO	Gene ontology
KEGG	Kyoto encyclopedia of genes and genomes
BP	Biological process
MF	Molecular function
CC	Cellular component
PPI	Protein–protein interaction
WB	Western blotting
FBS	Fetal bovine serum
PBS	Phosphate-buffered saline
TIC	Total ion chromatogram
PIP2	Phosphatidylinositol 3,4-bisphosphate
PIP3	Phosphatidylinositol 3,4,5-trisphosphate
